# Transcriptomic Analysis Reveals the Protective Effects of *Eucommia ulmoides* Leaf Extract Against D-Galactose-Induced Senescence in Avian Intestinal Epithelial Cells

**DOI:** 10.3390/foods15142526

**Published:** 2026-07-16

**Authors:** Xiaoxiao Liang, Yiru Cheng, Ruxia Wang, Qian Wang, Peng Tang, Yulong Yin, Xia Xiong

**Affiliations:** 1 College of Bioengineering, Henan University of Technology, Zhengzhou 450002, China; lxx2024@haut.edu.cn (X.L.); yrc@stu.haut.edu.cn (Y.C.); qianwang@stu.haut.edu.cn (Q.W.); 2 Henan Golden Lily Biotechnology Co., Ltd., Anyang 455000, China; 3 Institute of Biological Resources, Jiangxi Academy of Sciences, Nanchang 330096, China; wangruxia317@126.com; 4 Sanmenxia Ruizhiheng Pharmaceutical Co., Ltd., Sanmenxia 472200‌‌, China; 18503864888@163.com; 5 Institute of Subtropical Agriculture, Chinese Academy of Sciences, Changsha 410125, China; yinyulong@isa.ac.cn

**Keywords:** *Eucommia ulmoides* leaf extract, IECs, anti-aging, autophagy

## Abstract

*Eucommia ulmoides* leaf extract (ELE) boasts a high concentration of bioactive components including flavonoids, chlorogenic acid, and polysaccharides. It exhibits multiple biological functions, including antioxidant, anti-inflammatory, and gut microbiota-modulating properties, showing great potential in enhancing immunity, maintaining intestinal health, and delaying cellular senescence. This study investigated the protective effects and underlying mechanisms of ELE against D-galactose-induced senescence in chick embryo primary intestinal epithelial cells (IECs). Using an in vitro model (200 mmol/L D-galactose), we found that 100 µg/mL ELE pretreatment significantly preserved cell viability, mitigated apoptosis, and delayed cellular senescence, as evidenced by cytological and biochemical assays. Furthermore, RNA-seq transcriptomic analysis identified seven key differentially expressed genes (DEGs) mediating these anti-aging effects. Mechanistic investigations revealed that ELE modulates ATP6V0D2 and NCF2 to activate autophagy signaling pathways. This ELE-induced promotion of autophagy effectively suppresses inflammatory responses in IECs, thereby delaying senescence progression. These findings elucidate the molecular mechanisms by which ELE antagonizes intestinal cellular senescence, providing a solid theoretical foundation for its development as a functional anti-aging additive in the food industry.

## 1. Introduction

*Eucommia ulmoides* leaf extract (ELE) has earned a stellar reputation as a time-honored herbal cure-all and a prime component in health-conscious cuisine. It brims with bioactive substances, such as flavonoids, phenolics, terpenoids, and polysaccharides [1,2]. These ingredients pack a punch with a range of health benefits, including antioxidant, immune-balancing, cholesterol-lowering, blood sugar-reducing, inflammation-fighting, and cancer-blocking effects [3,4]. Notably, ELE has been proven safe for consumption, demonstrating significant efficacy in ameliorating lipid profiles and exerting robust antioxidant and antibacterial activities, thereby highlighting its immense potential as a functional dietary supplement [5].

Senescence involves the progressive shortening of telomeres, DNA damage, mitochondrial dysfunction, impaired autophagy, chronic inflammation, and dysregulated nutrient sensing. Consequently, mitigating age-related decline has become a focal point of contemporary research. Current anti-aging interventions primarily involve calorie restriction mimetics, autophagy inducers, senolytics, and NAD+ precursors [6,7,8,9]. Among these, natural antioxidant compounds—such as polyphenols, vitamins, and phytochemical extracts—play a crucial role in neutralizing oxidative stress, a primary driver of age-related cellular damage. Accumulating evidence suggests that *E. ulmoides* extracts exhibit pronounced anti-aging properties via multiple pathways. For instance, in Caenorhabditis elegans models, extracts from its leaves and male flowers have been shown to improve muscle function [10,11], maintain intestinal barrier integrity, and preserve mitochondrial homeostasis, ultimately extending both lifespan and healthspan. These effects are largely attributed to active components like chlorogenic acid and quercetin [12], which alleviate oxidative stress, enhance mitochondrial quality control, and modulate autophagy [13,14]. Furthermore, ELE exhibits anti-photoaging properties in skin models by scavenging reactive oxygen species (ROS), suppressing pro-inflammatory cytokines (e.g., IL-17), and promoting collagen synthesis via the TGF-β/Smad signaling pathway [15,16,17].

In the context of poultry production, laying hens entering the middle and late stages of their laying cycle undergo a comparable aging process, manifested as progressive oxidative stress accumulation, declining reproductive organ function, and reduced egg-laying performance [18]. These physiological changes mirror the hallmarks of biological aging observed in other model organisms. Given the established antioxidant and anti-aging properties of ELE, dietary supplementation with ELE may represent a promising strategy to attenuate age-related functional decline in laying hens, thereby sustaining egg production performance and improving overall flock productivity during the late laying period [19].

Recent studies emphasize that delaying aging is closely linked to maintaining gut microbiota homeostasis, reducing intestinal inflammation, and preserving autophagic flux [20,21,22]. Our previous research demonstrated that ELE effectively alleviates intestinal dysfunction in laying hens, maintains gut homeostasis, and prolongs the peak laying period, thereby improving both egg production yield and quality. However, the precise molecular mechanisms by which ELE mitigates intestinal cellular senescence, particularly its role in modulating apoptosis and autophagic signaling pathways, remain elusive. To address this gap, an in vitro cellular senescence model was established by treating primary chick embryo intestinal epithelial cells (IECs) with D-galactose. Utilizing this premature aging model, we systematically evaluated the potential anti-aging therapeutic benefits of ELE pretreatment. The protective effects and underlying mechanisms of ELE were comprehensively investigated through a combination of cellular functional assays, flow cytometry, and quantitative real-time PCR (qRT-PCR). Furthermore, RNA-Seq analysis was employed to pinpoint key differentially expressed genes (DEGs) and the associated signaling pathways, thereby elucidating the molecular network through which ELE mitigates IECs. The research seeks to illuminate the molecular processes responsible for ELE-induced mitigation of intestinal aging. Overall, this study aims to provide a robust theoretical foundation for the application of ELE as a natural functional additive to promote healthy aging. Furthermore, it is expected to lay the groundwork for future targeted interventions in age-related signal transduction pathways.

## 2. Materials and Methods

### 2.1. Preparation of ELE

*Eucommia* leaf extract was supplied by Zhangjiajie Hengxing Biotechnology Co., Ltd., (Zhangjiajie, Hunan, China) and prepared via aqueous extraction: *Eucommia* leaves were soaked for 2 h and then extracted twice with pure water at 75 °C for 30 min per extraction. The extract was allowed to settle for 12 h and then centrifuged (Eppendorf, Hamburg, Germany), filtered and concentrated under vacuum. The resulting concentrate was subjected to spray drying.

### 2.2. Isolation and Culture of Primary IECs from Chicken Embryos

This laboratory refined an in vitro isolation method for duodenal epithelial cells from broiler embryos, originally described by Ali A [23]. We used 100 18-day-old Hy-Line Gray layer embryos (supplied by Wen Zhen Chicken Farm, Jin xian, Nanchang City, Jiangxi Province, China). The primary IECs were cultured in DMEM/F12 (Gibco, Shanghai, China) containing 10% fetal bovine serum (Gibco, China) at 37 °C in a 5% CO_2_ incubator (Thermos, Kunshan, China) and could be passaged for up to 8 days.

#### 2.2.1. Establishment of a D-Gal-Induced IEC Senescence Model

A stock solution of D-gal (Aladdin, Shanghai, China) was made. The solution had a concentration of 1 mol/L. DMEM was used to dissolve the powder. IECs were placed into a 96-well plate. The seeding density was 1 × 10^4^ cells per mL. The medium was complete DMEM/F12. It had 10% fetal bovine serum added. Cells were grown at 37 °C. The incubator had 5% CO_2_. The cells were kept there for 24 h. Then the cells reached 80% confluence. After that, cells were given D-gal at different levels. The levels were 0, 100, 200, 300 and 400 mM. The cells were then left for 24 and 48 h. A Cell Counting Kit-8 (CCK-8) assay kit (UElandy, Suzhou, China) was used to check cell viability. For each well, 10 μL of CCK-8 reagent was added to 100 μL of serum-free DMEM/F12 medium. This mix was kept at 37 °C in the dark. It stayed there for 2 h. Then the liquid above the cells was taken and moved to a new 96-well plate. A plate reader was used to measure absorbance at 450 nm. Cell survival rate was worked out from that. The right D-gal concentration and time period were picked. This was done to build an aging model for IECs grown in the lab.

#### 2.2.2. The Effect of ELE on D-Gal-Induced IEC Aging

To investigate the protective effect of ELE against IEC senescence, cells were first pretreated with ELE at concentrations of 5, 25, 50, 100 and 200 µg/mL for 12 h. Subsequently, 200 mM D-galactosyl glucose was added to induce cellular senescence for a further 12 h. To determine the optimal ELE concentration, cell viability was assessed in each group using the CCK-8 assay. To ensure the efficacy of ELE, we concurrently employed β-galactosidase staining (Beyotime Biotechnology, Shanghai, China), β-galactosidase activity assays (Suzhou Keming Biotechnology Co., Ltd., Suzhou, China), flow cytometry (Agilent, Santa Clara, CA, USA), and qRT-PCR to evaluate ELE’s preventive and protective effects against cellular senescence and apoptosis.

##### CCK-8 Cell Viability Assay

ELE was dissolved in DMEM/F12 medium. Subsequently, ELE was added at concentrations of 5, 25, 50, 100 and 200 µg/mL to pre-treat IECs for 12 h. Subsequently, 200 mM D-galactose was added to each group for a further 12 h. Cell viability was assessed using the CCK-8 assay, and the optimal ELE concentration and treatment duration were determined based on cell survival rates.

##### Cell Senescence β-Galactosidase Staining Kit

This detection method employed β-galactosidase staining reagents to visualize the state of cellular senescence. With X-Gal as the substrate, cells treated with the senescence-specific β-galactosidase (SA-β-Gal) assay yielded blue–violet-stained cells. Consequently, cells or tissues exhibiting β-galactosidase activity appeared blue under ordinary optical microscopy. This reagent selectively stained senescent cells, failing to stain pre-senescent cells, quiescent cells, immortalized cells, or cancer cells. The procedure strictly adhered to the kit protocol.

##### β-Galactosidase Activity Assay

β-galactosidase serves as a biomarker for detecting cellular senescence. In this experiment, a β-galactosidase assay kit was used to measure β-galactosidase activity in senescent cells, strictly in accordance with the kit’s instructions for use.

##### Flow Cytometry Analysis of Apoptosis and Necrosis Rates

Flow cytometry was employed to assess apoptosis rates in the ELE-treated group, D-gal model group, and control group, thereby evaluating ELE’s effect on alleviating cellular senescence. Detection was performed using the Annexin V-FITC/PI (Beyotime Biotechnology, China) dual-fluorescent staining method. The fundamental principle of this assay is as follows: under normal conditions, phosphatidylserine (PS) resides within the cell membrane, whereas in senescent cells, PS becomes exposed on the cell surface. Annexin V-FITC, a calcium-binding protein with a molecular weight of 35–36 kD, specifically binds to phospholipids such as PS. PI is a nucleic acid dye that penetrates the cell membrane when cells are disrupted, such as during the metaphase of apoptosis or upon cell death, thereby staining the nucleus. Therefore, combining Annexin V-FITC and PI enables the differentiation of cells in early, late, or dead stages. The procedure follows the steps of the Annexin V-FITC/PI dual staining assay.

### 2.3. RNA-Seq Technology for IEC Transcriptome Analysis

#### 2.3.1. IEC RNA Extraction and Quality Control

Total RNA was isolated from primary intestinal epithelial cells (IECs) using a column-based extraction kit (HiPure Total RNA Mini Kit, R4121, Magen, Guangzhou, China) according to the manufacturer’s protocol with minor modifications. Briefly, cultured cells were washed twice with phosphate-buffered saline (PBS) and subsequently lysed in 1 mL of MagZol Reagent, followed by a 10 min incubation at room temperature to ensure complete homogenization. Phase separation was achieved by the addition of 200 μL chloroform, vigorous mechanical shaking for 15 s, and a 3 min incubation at room temperature, after which samples were centrifuged at 12,000× *g* for 15 min at 4 °C. The upper aqueous phase, containing total RNA, was carefully collected and mixed with an equal volume of isopropanol to facilitate RNA precipitation. The resulting mixture was transferred to a HiPure RNA MiniColumn, followed by centrifugation and sequential washing with RW2 buffer to remove residual impurities. RNA was finally eluted with 50 μL of RNase-free water.

RNA integrity and quality were assessed using two complementary approaches: electrophoresis on a 1% TAE agarose gel and capillary electrophoresis on an Agilent 2100 Bioanalyzer, from which the RNA Integrity Number (RIN) was derived. RNA purity was evaluated spectrophotometrically using a NanoDrop spectrophotometer by measuring the OD_260_/_280_ and OD_260_/_230_ absorbance ratios. RNA concentration was quantified using a Qubit 2.0 Fluorometer. All RNA samples were stored at −80 °C until further use.

#### 2.3.2. Library Construction and Sequencing

Total RNA was turned into cDNA. This step is called reverse transcription. Only good-quality samples were used to build a cDNA library. Primers were designed with Primer 5.0. Sangon Biotech (Shanghai, China) (all primer sequences are listed in Table 1).

#### 2.3.3. Analysis of Differentially Expressed Genes (DEGs)

Gene expression differences were analyzed based on Fragments Per Kilobase of transcript per Million mapped reads (FPKM) values using the DESeq2 R package (v1.14.1). The Benjamini–Hochberg method was applied to adjust *p*-values to control the false discovery rate (FDR). Genes meeting the criteria of FPKM > 1, adjusted *p* (*p*adj) < 0.05, and |log2FoldChange| > 1 were defined as differentially expressed genes (DEGs). Hierarchical clustering and heatmap generation were performed using the pheatmap package in R.

#### 2.3.4. GO and KEGG Enrichment Analysis

Kyoto Encyclopedia of Genes and Genomes (KEGG) pathway enrichment and Gene Ontology (GO) annotation for DEGs were performed using KOBAS (v2.0) software. Terms and pathways with *p* < 0.05 were considered significantly enriched.

### 2.4. Protein–Protein Interaction (PPI) Network Analysis

The interaction network of differentially expressed proteins was constructed using the STRING database (http://string-db.org). Diamond alignment was applied to map the target gene sequences to protein sequences of reference or closely related species in the STRING database. The resulting interaction data was imported into Cytoscape 3.9.1 software to visualize the PPI network.

### 2.5. Statistical Analysis

Statistical analyses were performed using SPSS 19.0 software. Data are presented as the mean ± standard error (SE). Differences between groups were evaluated using one-way analysis of variance (ANOVA) followed by independent samples *t*-tests. A *p*-value < 0.05 was considered statistically significant, and *p* < 0.01 was considered highly significant. Graphs were generated using GraphPad Prism 8.

## 3. Results

### 3.1. Isolation and Culture of IECs

**Figure 1:**
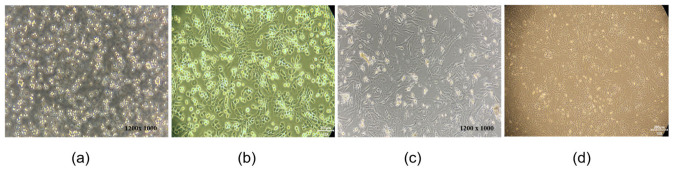
Isolated and cultured IECs isolated and cultured: (**a**) 0 h; (**b**) 24 h; (**c**) 48 h; (**d**) 96 h.

To establish an in vitro model for investigating the effects of external nutrients on the intestinal epithelium, primary IECs were successfully isolated from the duodena of 18-day-old chicken embryos. The morphological progression of the IEC cultures at 0, 24, 48, and 96 h post-isolation is depicted in Figure 1a–d.

### 3.2. Establishment of a D-Gal-Induced IEC Senescence Model

**Figure 2:**
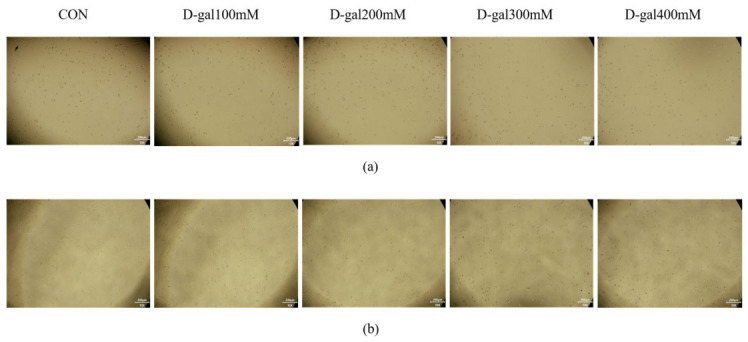
D-gal-induced cellular senescence model. Morphological observation of cells treated with D-gal and CCK-8 cell viability assay: (**a**) 24 h after D-gal treatment; (**b**) 48 h after D-gal treatment.

To determine the optimal D-gal concentration and exposure time for inducing IEC senescence, cells were treated with 0, 100, 200, 300, and 400 mM D-gal for 24 h and 48 h. Morphological observation (Figure 2a,b) revealed that after 24 h of D-gal treatment, IECs exhibited enlarged cell volumes, widened intercellular gaps, and reduced cell proliferation compared to the control group, indicating the onset of senescence. After 48 h of exposure, the cells displayed severe senescence and apoptosis, characterized by cellular fragmentation and a significant reduction in cell mass.

**Figure 3:**
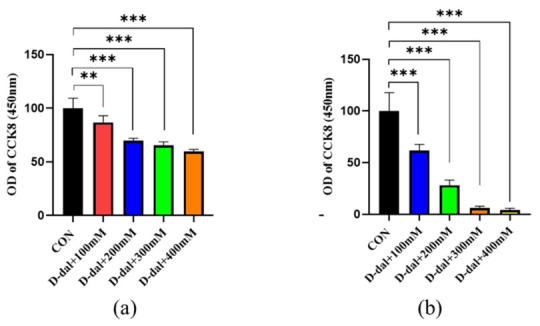
CCK-8 cell viability assay: (**a**) 24 h cell viability; (**b**) 48 h cell viability. * Denotes significant difference (*p* < 0.05), and ** denotes very significant difference (*p* < 0.01), and ***denotes extremely significant difference (*p* < 0.001).

As shown in Figure 3a,b, IEC viability decreased significantly across all D-gal concentrations after 24 h (*p* < 0.01). Notably, viability exhibited a highly significant decline (*p* < 0.001) at 200 mM D-gal. Following 48 h of treatment, cell viability in all D-gal groups was drastically reduced compared to the control (*p* < 0.001), dropping below 10% in the 300 mM and 400 mM groups. Based on the CCK-8 assay and morphological observations, exposure to 200 mM D-gal for 24 h induced a stable state of cellular senescence. Thus, this condition was selected for subsequent experiments.

### 3.3. Effect of ELE on D-Gal-Induced Senescence in IECs

**Figure 4:**
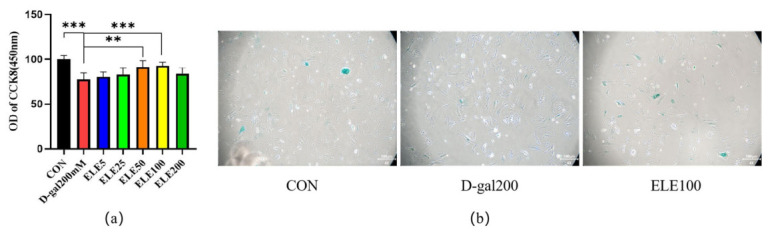
D-gal induced cellular senescence model and the effects of ELE intervention. (**a**) CCK-8 cell viability assay; (**b**) observation of cell morphology. * Denotes significant difference (*p* < 0.05), and ** denotes very significant difference (*p* < 0.01), and ***denotes extremely significant difference (*p* < 0.001).

To assess the protective effects of ELE against D-gal-induced senescence, IECs were pretreated with various concentrations of ELE (5–200 µg/mL) for 12 h prior to a 12 h induction with 200 mM D-gal. As depicted in Figure 4a, pretreatment with 50 µg/mL and 100 µg/mL ELE significantly restored cell viability compared to the D-gal model group (*p* < 0.01). While the 200 µg/mL ELE group also showed improved viability, the difference was not statistically significant. These results suggest that ELE effectively counteracts D-gal-induced cytotoxicity, with 100 µg/mL identified as the optimal concentration.

To further validate these anti-senescence effects, SA-β-gal staining was performed. As shown in Figure 4b, the control group exhibited dense, actively dividing cells with minimal staining. In contrast, the D-gal group displayed abundant blue staining, with cells presenting enlarged, flattened morphologies, numerous vacuoles, and hypertrophic nuclei. In the ELE pretreatment group, staining was notably sparser, and cells maintained a more compact morphology, indicating a reduced proportion of senescent cells. Consistent with the staining results, biochemical assays (Table 2) showed that while D-gal treatment elevated intracellular β-galactosidase activity relative to the control, the addition of 100 µg/mL ELE reversed this trend, decreasing β-galactosidase activity in senescent IECs.

**Figure 5:**
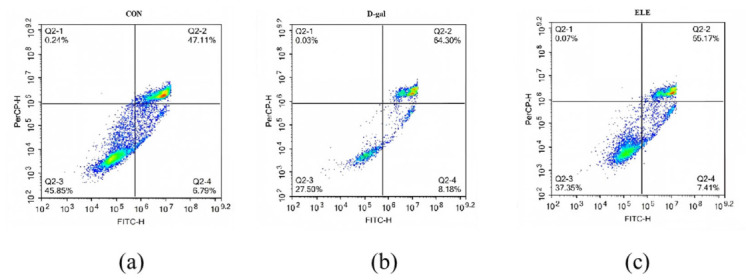
Effect of ELE on apoptosis and necrosis of D-gal-induced IECs. (**a**) CON; (**b**) D-gal; (**c**) ELE. Note: Blue, green, and yellow/red indicate low, moderate, and high cell densities, respectively.

Flow cytometry was utilized to evaluate the impact of ELE on D-gal-induced apoptosis. As presented in Figure 5, Compared with the CON group, the proportion of late apoptotic/necrotic cells in the D-gal group increased significantly (+17.19%), while the proportion of normal viable cells decreased substantially (−18.35%), indicating that D-gal successfully induced cellular senescence. Following ELE treatment, the proportion of late apoptotic/necrotic cells decreased by(−9.13%), and the proportion of normal viable cells recovered by (+9.85%). As shown in Table 3, the apoptosis rate in the D-gal 200 group was significantly elevated compared to that in the control group (increased by 22.2%, *p* < 0.05). Pretreatment with 100 µg/mL ELE reduced the apoptosis rate by 12.6% compared to that of the D-gal group, suggesting that ELE exerts an inhibitory effect on D-gal-induced apoptosis in IECs.

**Figure 6:**
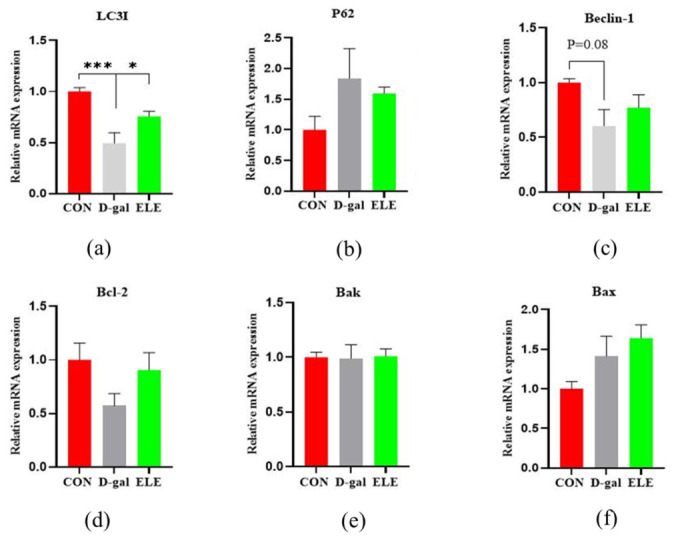
Effects of ELE on the mRNA expression of autophagy- and apoptosis-related genes in D-galactose-induced IECs. Relative mRNA expression levels of (**a**) LC3I, (**b**) P62, (**c**) Beclin-1, (**d**) Bcl-2, (**e**) Bak1, and (**f**) Bax were determined by RT-qPCR. * Denotes significant difference (*p* < 0.05), and *** denotes extremely significant difference (*p* < 0.001).

To elucidate the underlying molecular mechanisms, the mRNA expression levels of autophagy-related factors (LC3I, P62, Beclin-1) and apoptosis-related factors (Bcl-2, Bak, Bax) were measured via qRT-PCR (Figure 6). Compared to the control group, LC3I was significantly downregulated in the D-gal group, an effect that was significantly reversed by ELE treatment. Furthermore, Beclin-1 and the anti-apoptotic gene Bcl-2 were downregulated in the D-gal group but upregulated following ELE treatment, although these changes did not reach statistical significance. The expression of the pro-apoptotic genes Bak and Bax showed no significant differences across the groups. Collectively, these findings imply that ELE mitigates D-gal-induced IEC senescence, at least in part, by preserving autophagic activity and suppressing apoptosis.

### 3.4. Transcriptome Analysis of IECs via RNA-Seq

#### 3.4.1. RNA Sequencing Data Quality

**Figure 7:**
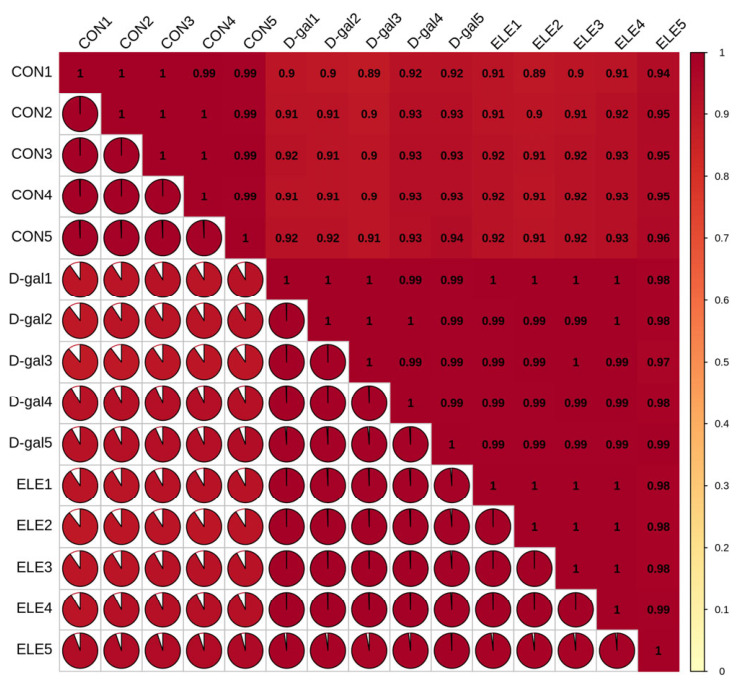
Pearson correlation between the 15 samples.The color bar on the right indicates correlation strength: the darker and redder the color, the closer the correlation coefficient is to 1, implying a stronger correlation.

Illumina HiSeq sequencing of 15 libraries (CON 1–5, D-gal 1–5, and ELE 1–5) generated a total of 110.1 Gb of clean data. All samples yielded >6 Gb of high-quality data, with Q30 percentages exceeding 92%. Clean reads were aligned to the reference genome (Gallus.GRCg6a.dna.toplevel.fa), resulting in 77.39–79.64% mapping to exons, 13.74–14.95% to intergenic regions, and 6.62–7.68% to introns. The GC content of the clean reads ranged from 48.04% to 48.48%. As shown in Figure 7, Pearson’s correlation coefficients (R^2^) among biological replicates were all >0.8, demonstrating high reproducibility and reliability for subsequent DEG analysis.

**Figure 8:**
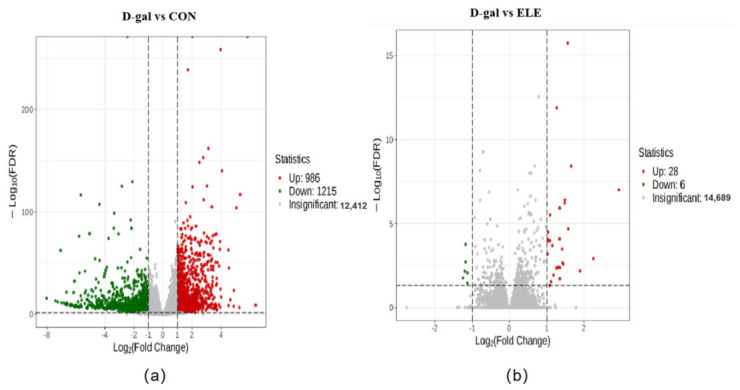
Volcano plot for differential expression analysis of the RNA-seq transcriptome. (**a**) Volcano diagram of differentially expressed genes D-gal VS CON; (**b**) volcano diagram of differentially expressed genes D-gal VS ELE. Note: DEGs are shown as green dots indicating downregulation and red dots indicating upregulation, while gray dots indicate no significance.

A total of 22,935 genes were identified, comprising 21,042 known genes and 1893 novel genes. Under the threshold of *p* < 0.05 and |log2FoldChange| ≥ 1, 2612 DEGs were screened. Specifically, 2201 DEGs were identified in the D-gal vs CON comparison (986 upregulated, 1215 downregulated; Figure 8a), while 34 DEGs were found in the D-gal vs ELE comparison (6 upregulated, 28 downregulated; Figure 8b).

#### 3.4.2. Identification and Clustering of DEGs

**Figure 9:**
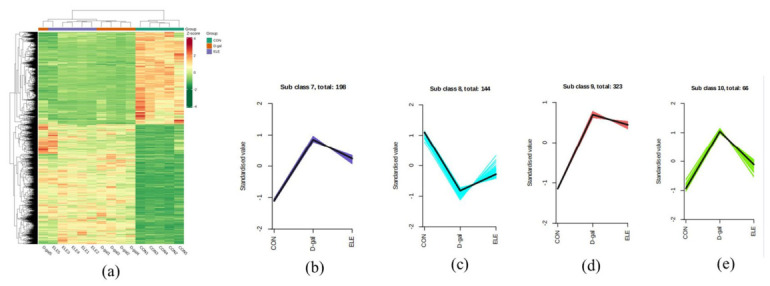
Transcriptome sequencing analysis. (**a**) Cluster analysis of DEG among of three groups by the FPKM value; (**b**–**e**) Kmeans cluster analysis of the three groups of DEG based on FPKM values. Note(a): Highly expressed genes are shown in red and low expressed genes are shown in green. The closer the branch distance between the two samples, the more similar the expression pattern and change trend of all genes in the two samples. Note(b): The horizontal coordinate represents the sample, and the vertical coordinate represents the FPKM normalized expression of the same gene.

Hierarchical clustering (Figure 9a) revealed distinct expression patterns between the ELE and D-gal groups, implying divergent metabolic processes and pathway activations. K-means clustering (Figure 9b–e) further confirmed that the CON and ELE groups shared similar gene expression trends, which were starkly opposite to those observed in the D-gal group, suggesting that ELE treatment restores normal functional characteristics in IECs.

**Figure 10:**
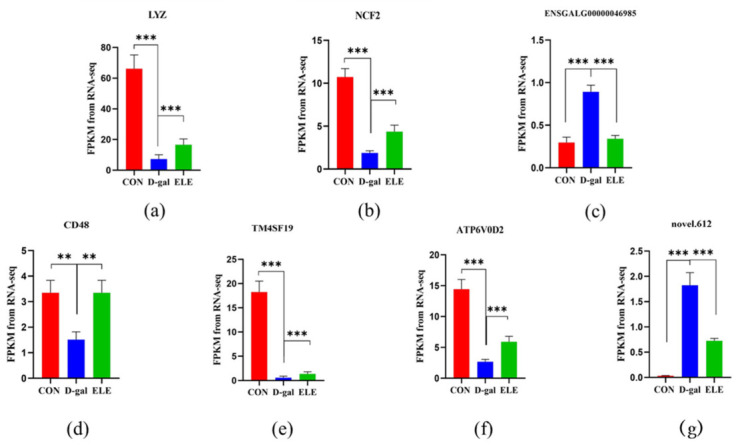
Effects of ELE on the mRNA expression of immune- and lysosome-related genes in D-galactose-induced IECs. FPKM values of (**a**) LYZ, (**b**) NCF2, (**c**) ENSGALG00000046985, (**d**) CD48, (**e**) TM4SF19, (**f**) ATP6V0D2, and (**g**) novel.612 are shown. ** Denotes very significant difference (*p* < 0.05), and *** denotes extremly significant difference (*p* < 0.001).

To pinpoint the key genes regulated by ELE, we intersected the DEGs from the D-gal vs. ELE and D-gal vs. CON comparisons. Of the 34 DEGs in the D-gal vs. ELE group, 27 were also differentially expressed in the D-gal vs. CON group. Among these, seven core DEGs exhibited exactly opposite regulatory patterns between the two comparisons (e.g., upregulated by D-gal but downregulated by ELE). As shown in Figure 10, the FPKM expression trends of these seven key genes were highly consistent between the CON and ELE groups but opposite in the D-gal group.

#### 3.4.3. KEGG Pathway Enrichment Analysis of DEGs

**Figure 11:**
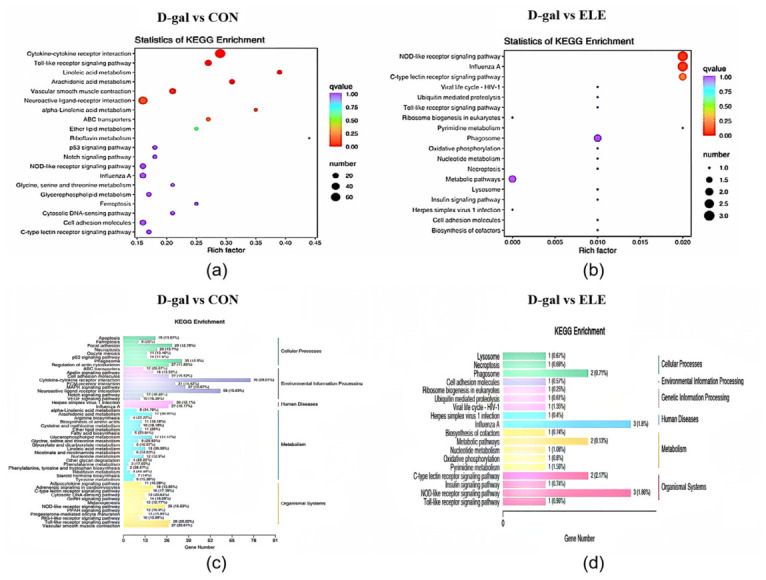
KEGG pathway enrichment analysis. (**a**) D-gal_vs_CON bubble plot; (**b**) D-gal_vs_ELE bubble plot; (**c**) D-gal_vs_CON bar chart; (**d**) D-gal_vs_ELE bar chart.

To elucidate the signaling pathways mediating the anti-senescence effects of ELE, KEGG enrichment analysis was performed. DEGs were mapped to 319 KEGG pathways. The top 20 enriched pathways for D-gal vs. CON and ELE vs. D-gal are presented in Figure 11a,b.

As shown in Figure 11c,d, DEGs from the D-gal vs. CON comparison were significantly enriched in cellular processes, metabolism, environmental information processing, organismal systems, and human diseases. Specifically, within cellular processes, DEGs were highly associated with phagocytosis (35 genes), cell adhesion (29 genes), and regulation of the actin cytoskeleton (27 genes). Environmental information processing was predominantly driven by cytokine–cytokine receptor interactions (76 genes), neuroactive ligand–receptor interactions (58 genes), and the MAPK signaling pathway (37 genes). Metabolic enrichment was led by arachidonic acid (37 genes), glycerophospholipid (17 genes), and linoleic acid metabolism (13 genes). Finally, significant enrichment was observed in immune and organismal system pathways, including Toll-like receptor signaling (28 genes), vascular smooth muscle contraction (27 genes), and NOD-like receptor signaling (25 genes).

D-gal_vs_ELE differentially expressed genes were predominantly enriched across five categories: cellular processes, metabolism, genetic information processing, organismal systems, human diseases, and environmental information processing. Key pathways included the phagosome signal pathway, metabolic pathways, ubiquitin-mediated proteolysis, influenza A signal pathway, NOD-like receptor signal pathway, C-type lectin receptor signal pathway, and cell adhesion molecule signal pathway.

To further elucidate how ELE suppresses D-gal-induced IEC apoptosis via differential expression of genes, we analyzed KEGG pathways for seven key differentially expressed genes. As shown in Table 4 five differential genes lacked annotation to KEGG pathways. ENSGALG00000004700.6 (NCF2) was predominantly enriched in the phagosome pathway, while ENSGALG00000034294.2 (ATP6V0D2) showed significant enrichment in oxidative phosphorylation, metabolism, lysosome, and phagosome pathways.

#### 3.4.4. qRT-PCR Validation of DEGs Identified by RNA-Seq

**Figure 12:**
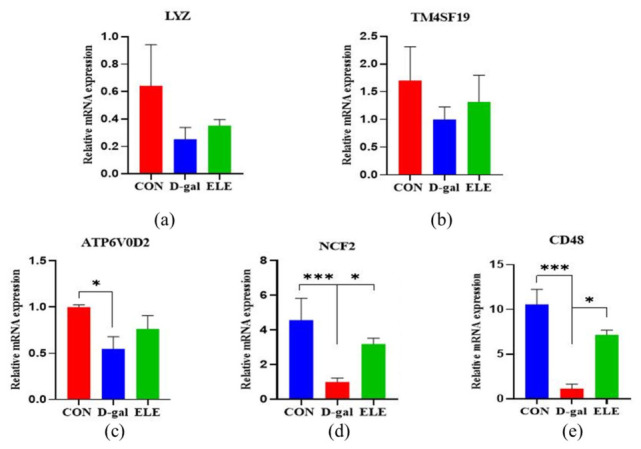
qRT-PCR confirmed the results of RNA-seq differentially expressed genes. (**a**) LYZ; (**b**) TM4SF19; (**c**) ATP6V0D2; (**d**) NCF2; (**e**) CD48. * Denotes significant difference (*p* < 0.05), and *** denotes extremly significant difference (*p* < 0.001).

qRT-PCR validated the mRNA expression of five differentially expressed genes in IECs. As shown in Figure 12, compared with the CON group, the ATP6V0D2 gene mRNA expression was significantly downregulated in the D-gal group (*p* < 0.05). Compared with the D-gal group, the ATP6V0D2 gene expression was slightly upregulated in the ELE group, though the difference was not statistically significant. Compared with the control group, mRNA expression of the NCF2 and CD48 genes was significantly downregulated (*p* < 0.01). Compared with the D-gal group, mRNA expression of both NCF2 and CD48 genes was significantly upregulated in the ELE group. Therefore, we found that ELE pretreatment reversed the downregulation of ATP6V0D2, NCF2, CD48, LYZ, and TM4SF19 in the D-gal group, with the ELE group exhibiting mRNA expression patterns consistent with the CON group. Results indicate that qRT-PCR findings for differentially expressed genes align with RNA-seq sequencing data, validating the reliability of the RNA-seq differential expression profiling method and confirming the authenticity and validity of the data for subsequent analysis.

### 3.5. Protein–Protein Interaction (PPI) Network Analysis

**Figure 13:**
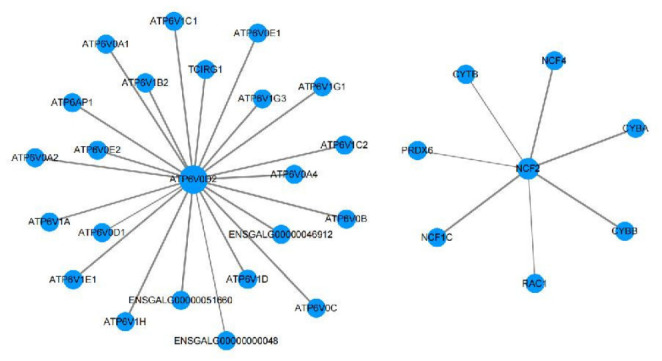
Module diagram of the protein interaction network. In the network diagram, genes are represented by circles, the size of the graph indicates the size of the connectivity, and the thickness of the line indicates combined score.

To systematically investigate gene interactions, the seven core DEGs and all genes within their associated signaling pathways were mapped using Cytoscape. Utilizing the MCODE plugin, two significant functional modules were identified (Figure 13). Further screening based on the Degree algorithm in the CytoHubba plugin revealed that ATP6V0D2 and NCF2 serve as the core hub genes within these networks.

## 4. Discussion

With rising living standards, global demand for animal-sourced foods—particularly eggs, meat, and dairy—has grown substantially. As one of the most important sources of high-quality protein, eggs occupy a central role in daily diets worldwide, especially in China. However, following peak egg production, laying hens progressively enter the middle and late stages of their laying cycle, during which physiological aging leads to a significant decline in egg-laying performance. Given that this phase accounts for approximately half of the total production period, mitigating age-related performance deterioration represents a critical bottleneck for the economic efficiency of the poultry industry [24,25,26]. In this context, the use of feed additives has emerged as a practical and cost-effective strategy to attenuate the decline in laying performance. Bioactive compounds such as antioxidants, phytogenic additives, and prebiotics have been shown to alleviate oxidative stress, modulate gut microbiota, and support reproductive organ function in aging hens, thereby helping to maintain egg production rate, egg quality, and feed conversion efficiency during the late laying period [27]. The findings of the present study are consistent with this body of evidence, suggesting that targeted dietary supplementation may offer a viable approach to extending productive longevity and improving the overall economic output of laying hen flocks [28].

To mitigate these degenerative changes, the molecular mechanisms underlying the anti-aging properties of natural bioactive compounds have garnered considerable attention in recent years. Phytochemicals have been shown to regulate cellular lifespan via post-translational histone modifications and the upregulation of autophagy [24]. Numerous natural compounds, such as resveratrol, astaxanthin, and gallic acid, exhibit the capacity to delay biological aging and extend lifespan [19,29,30]. To date, over 300 compounds or clinical drugs have been reported to possess anti-aging activities, among which 185 are plant-derived [31]. For instance, chlorogenic acid derived from Eucommia ulmoides has demonstrated significant lifespan-extending effects in a Caenorhabditis elegans model [32]. Eucommia is a traditional medicinal plant endemic to China. Over 200 active compounds have been isolated from it, 86 of which are concentrated in its leaves, primarily including lignans, iridoids, phenolic acids, flavonoids, and polysaccharides [24]. Due to its rich composition of these bioactive constituents, Eucommia leaf extract (ELE) exerts broad pharmacological effects, including antibacterial, anti-inflammatory, antioxidant, antitumor, immunomodulatory, and anti-aging properties [25]. Therefore, elucidating the regulatory pathways through which ELE inhibits intestinal senescence holds significant theoretical and practical value for poultry farming.

Previous in vivo studies have suggested that ELE mitigates the decline in egg-laying performance in aging hens by modulating the gut microbiota and serum metabolome [19]. To further validate these findings and elucidate the cellular mechanisms by which ELE alleviates intestinal aging, the present study utilized primary intestinal epithelial cells (IECs) isolated from chicken embryos as an in vitro model. The isolation and primary culture of IECs provide an efficient and physiologically relevant platform for investigating intestinal biological functions, nutrient absorption, and epithelial barrier regulation [26]. Ali et al. [23] successfully established a material absorption model using primary chicken embryo duodenal epithelial cells. Because primary cells retain the physiological and genetic characteristics of their tissue of origin better than immortalized cell lines, they are considered superior in vitro models for pharmacological and nutritional screening. Therefore, utilizing primary chicken IECs to investigate the protective effects of ELE against senescence is scientifically robust.

The D-galactose (D-gal)-induced aging model is a widely recognized in vitro tool for senescence research [29]. While D-gal-induced accelerated aging has been successfully established in various cell types—including neural stem cells, astrocytes, mesenchymal stem cells, HUVECs, NRK-52E cells [30,31,32,33,34], and, recently, porcine IECs [35]—no studies have yet reported its application in avian IECs. In the current study, we successfully established a senescence model by exposing primary chicken IECs to 200 mM D-gal for 24 h. This novel in vitro avian intestinal aging model lays the foundation for investigating the mechanisms of ELE in mitigating gut senescence in laying hens.

Building upon this model, we evaluated the cytoprotective effects of ELE. CCK-8 assays revealed that ELE pretreatment dose-dependently enhanced cell viability and counteracted D-gal-induced cytotoxicity, peaking at a concentration of 100 µg/mL. Interestingly, the protective efficacy diminished at 200 µg/mL. This may be partly attributed to the limited metabolic capacity of primary IECs, where an excessive influx of exogenous substances could inadvertently increase intracellular metabolic burden [36]. Furthermore, SA-β-gal staining and activity assays demonstrated that 100 µg/mL ELE significantly reduced both the proportion of senescent cells and β-galactosidase activity. Flow cytometric analysis further corroborated these findings, showing a 12.6% reduction in the apoptosis rate following ELE pretreatment. These results align with a previous study by Wang et al. [13], which reported that specific Eucommia components mitigated H_2_O_2_-induced oxidative stress, regulated the cell cycle, and inhibited cellular senescence via multiple coordinated pathways. At the molecular level, our qRT-PCR analysis revealed that D-gal significantly downregulated the expression of the autophagy marker LC3I, an effect that was effectively reversed by ELE pretreatment. Similarly, Lai et al. [37] reported that Eucommia-derived quercetin upregulated autophagy-related proteins (LC3, Atg5, Beclin-1) to alleviate renal fibrosis in a high-glucose-induced model. Additionally, Dong et al. [32] demonstrated that restoring autophagic flux delayed ovarian aging in hens. Based on these collective findings, we deduce that ELE mitigates D-gal-induced IEC senescence by preserving cell viability, lowering β-galactosidase activity, suppressing apoptosis, and restoring autophagic flux.

To comprehensively map the molecular networks governing these protective effects, we performed transcriptomic analysis (RNA-Seq) on the CON, D-gal, and ELE groups. By comparing the expression profiles, we identified seven key differentially expressed genes (DEGs) (LYZ, NCF2, ATP6V0D2, CD48, TM4SF19, ENSGALG00000046985, novel.612) that exhibited inverse regulatory patterns between the D-gal_vs_CON and ELE_vs_D-gal comparisons. KEGG enrichment analysis revealed that NCF2 and ATP6V0D2 were significantly enriched in autophagic, lysosomal, and oxidative phosphorylation pathways. Autophagy is a critical degradation mechanism essential for clearing damaged organelles and long-lived proteins, thereby preventing inflammasome hyperactivation [38,39]. Abundant evidence indicates that robust autophagic activity is intrinsically linked to delayed aging. For instance, enhanced autophagy extends lifespan and prevents age-related proteinopathy in C. elegans [40], and the overexpression of Atg5 in mice confers resistance to oxidative stress and prolongs survival [41].

Crucially, the age-related decline in autophagic efficacy is often driven by impaired lysosomal function [42]. Vacuolar ATPase (V-ATPase) is a proton pump indispensable for maintaining lysosomal acidification and homeostasis. ATP6V0D2, encoding the d2 subunit of V-ATPase, is critical for autophagosome–lysosome fusion. Recent studies indicate that ATP6V0D2 interacts with the SNARE complex (STX17 and VAMP8) to facilitate this fusion, a step vital for completing autophagic degradation and limiting inflammation [43,44]. Macrophages lacking ATP6V0D2 accumulate damaged mitochondria and exhibit excessive inflammasome activation [44]. In our study, D-gal treatment significantly downregulated ATP6V0D2 expression, which was effectively restored by ELE supplementation. Consequently, we postulate that ELE restores autophagic flux in senescent IECs by upregulating ATP6V0D2, thereby promoting autophagosome–lysosome fusion, clearing intracellular detritus, and mitigating intestinal inflammation.

Another critical hub gene identified was NCF2 (encoding p67phox), an essential cytosolic subunit of the NADPH oxidase 2 (NOX2) complex. NCF2 is required for the generation of reactive oxygen species (ROS) that drive innate immune defenses [45,46]. Beyond pathogen clearance, tightly regulated NCF2-mediated ROS production exerts anti-apoptotic effects, supporting cell survival and modulating oxidative stress homeostasis [47,48]. Interestingly, NCF2 expression is uniquely regulated by the tumor suppressor p53 [49], linking it directly to cellular stress responses. In the present study, NCF2 was significantly suppressed in the D-gal-induced senescence model but dramatically upregulated following ELE treatment. We hypothesize that D-gal-induced senescence impairs NOX2 assembly by downregulating NCF2, leading to compromised innate immune signaling in the gut. Conversely, ELE may activate p53-dependent or independent pathways to rescue NCF2 expression. This restoration likely normalizes basal NADPH oxidase activity and maintains redox signaling homeostasis, thereby preventing apoptosis, bolstering innate intestinal immunity, and ultimately protecting the gut barrier against age-related decline.

## 5. Conclusions

In summary, this study successfully established a novel in vitro senescence model using primary IECs isolated from chicken embryos, induced by exposure to 200 mM D-galactose for 24 h. Comprehensive evaluations—including CCK-8 assays, β-galactosidase staining, flow cytometry, and qRT-PCR—demonstrated that pretreatment with 100 µg/mL ELE effectively mitigates D-gal-induced cellular senescence by inhibiting apoptosis and restoring autophagic flux. Furthermore, transcriptomic analysis via RNA-Seq identified a total of 2610 DEGs. Through comparative analysis across the experimental groups, seven core DEGs were pinpointed, with NCF2 and ATP6V0D2 emerging as pivotal hub genes. KEGG enrichment analysis revealed that NCF2 is primarily involved in phagosomal signaling, while ATP6V0D2 is significantly enriched in oxidative phosphorylation, lysosomal function, and phagosome pathways. Based on these findings, we conclude that ELE upregulates ATP6V0D2 and NCF2 to synergistically activate autophagic and innate immune pathways. This coordinated molecular response facilitates the clearance of cellular debris, suppresses intestinal inflammation, and maintains gut homeostasis, ultimately delaying cellular aging. These results provide profound mechanistic insights and highlight the immense potential of ELE as a natural, multifunctional food additives in the food industry to alleviate age-related intestinal dysfunction and improve product quality, safety, and the healthiness of natural foods.

## Figures and Tables

**Table 1:** qRT-PCR specific primer sequences.

Target Gene	Accession Number	Synthesized Sequence 5′-3′	Prod Size (bp)
LC3I	XM_417327.5	F: TTACACCCATATCAGATTCTTG R: ATTCCAACCTGTCCCTCA	143
Beclin-1	NM_001006332.1	F: CGTATGGCAACCACTCGTATT R: TTATTGTCCCAGAAGAACCTCAG	97
P62	NC_006100.4	F: GACCCAGCCAAGACTACCAT R: CAGAGGCATGTAGTTTCGGC	239
Bak1	NM_001030920.1	F: ATGGATGCCTGTCTGTCCTGTTC R: GCAGAGCAGTCCAAAGACACTGA	106
Bax	XM_015290061.1	F: TCCTCATCGCCATGCTCAT R: CCTTGGTCTGGAAGCAGAAGA	69
Bcl-2	NM_205339.2	F: GATCGTCGCCTTCTTCGAGT R: GGCCTCATACTGTTGCCGTA	186
GAPDH	NM_204305.2	F: CTAGAGAGGCTGGGCAAGGAA R: CTCTGAGTGAACGCCTCCTCTG	244
NCF2	XM_004943277.5	F: GGTTGACACGCCATTAGCTG R: CGTCTGTCTGCCTCAAACTCA	286
ATP6V0D2	NM_001008455.2	F: GCCCACCTGTGGCAAATTGA R: GGCAGACGAGAAGTAAAGACCAA	192
TM4SF19	XM_ 040705551.2	F: ACCGTAACACCGATGGGAGTACC R: AGTTCACGCTCGCTGTTATGTCTG	287
LYZ	NM_205281.2	F: GTGGTGGAGGACCGCAAGCC R: GGGTTGAAGGAGACGGACAGC	150
CD48	NM_001318404.1	F: CTAGAGAGGCTGGGCAAGGAA R: CTCTGAGTGAACGCCTCCTCTG	140
GAPDH	NM_204305.2	F: CTAGAGAGGCTGGGCAAGGAA R: CTCTGAGTGAACGCCTCCTCTG	244

Paired-end sequencing was performed on the Illumina platform. Transcript assembly was carried out using StringTie version 1.3.4d. Novel gene identification was also conducted with this tool. FeatureCounts version 1.6.2 was used to calculatework out gene alignment counts.

**Table 2:** Effect of ELE on β-GAL activity of D-gal-induced IECs.

Item	Control	D-Gal 200 mM	ELE100 µg/mL	SEM	*p*-Value
β-GAL activity	116.05 ± 8.06	145.71 ± 31.07	113.57 ± 17.09	11.88	0.50

**Table 3:** Effect of ELE on apoptosis and necrosis of D-gal-induced IECs.

Item	Control	D-Gal 200 mM	ELE 100 µg/mL	SEM	*p*-Value
Cell apoptosis rate, %	49.71 ± 3.05 b	60.77 ± 4.24 a	55.43 ± 0.15 ab	2.20	0.11

Note: Different lowercase letters within the same row indicate significant differences (*p* < 0.05); values sharing the same superscript letter are not significantly different *(p* > 0.05).

**Table 4:** KEGG signaling pathways enriched by 7 differential genes.

Gene id	Gene Name	ELE_Count	D-Gal_Count	*p*-Value	Ko id	KEGG Pathway
ENSGALG00000009963.5	LYZ	181	70.2	2.80028 × 10^−7^	K13915	--
ENSGALG00000004700.6	NCF2	225.4	83.8	4.01946 × 10^−7^	K08010	ko04145: phagosome
ENSGALG00000046985.1	ENSGALG000 00046985	13	30.4	0.000	--	--
ENSGALG00000035856.2	CD48	112.8	44.4	7.55747 × 10^−5^	K06479	--
ENSGALG00000006375.6	TM4SF19	87.2	31.4	0.000780459	K24922	--
ENSGALG00000034294.2	ATP6V0D2	194	78	4.00055 × 10^−6^	K02146	ko00190: oxidative phosphorylation; ko01100: metabolic pathways; ko04142: lysosome; ko04145: phagosome
novel.612	--	83.4	183.2	4.53299 × 10^−10^	--	--

Note: “--” indicates unannotated or unavailable data: Gene Name: No official gene symbol assigned (e.g., novel transcript). Ko id: Unmapped to KEGG Orthology. KEGG Pathway: Not assigned to any known KEGG pathway.

## Data Availability

The original contributions presented in the study are included in the article; further inquiries can be directed to the corresponding author.
